# Regaining power: How feelings of exclusion during COVID-19 are associated with radicalism among critics of containment policies

**DOI:** 10.3389/fpsyg.2022.952760

**Published:** 2022-10-28

**Authors:** Michaela Pfundmair, Luisa A. M. Mahr

**Affiliations:** ^1^Faculty of Intelligence, Federal University of Administrative Sciences, Berlin, Germany; ^2^Faculty of Psychology, Alpen-Adria University of Klagenfurt, Klagenfurt, Austria

**Keywords:** social exclusion, COVID-19, radicalism, control, power

## Abstract

Past experimental research has shown that social exclusion can be linked with radicalism. During the COVID-19 pandemic, feelings of social isolation and loneliness rose, just like protests and violence against national anti-COVID-19 measures did. Based on these observations, we hypothesized that feelings of exclusion induced by measures to contain the spread of COVID-19 were associated with radicalism intentions to illegally and violently fight COVID-19-related regulations among critics of the containment policies (Hypothesis 1). Moreover, we expected that radicalism intentions against COVID-19-related regulations fortified needs deprived by social exclusion (Hypothesis 2). Studying a sample of individuals who opposed the measures to contain the spread of COVID-19 (*N* = 171), we found evidence for both hypotheses: Results revealed that feelings of social exclusion induced by COVID-19 containment measures predicted radicalism intentions. Moreover, the relationship between exclusion and radicalism was associated with fortifying power issues. Political opinion did not moderate these effects. These data replicate the exclusion-radicalism link in the COVID-19 crisis and add one more factor that may have promoted radical developments during that time. Fortifying feelings of power, radicalism appeared to foster well-being, though at a high political price.

## Introduction

With the appearance of COVID-19, another phenomenon rose: radicalism, the readiness to engage in illegal and violent political action (Moskalenko and McCauley, 2009). This could not only be observed among, in particular, right-wing extremist groups that capitalized on COVID-19 to spread disinformation that scapegoated marginalized groups and endorsed instances of violence (Davies, 2021). Also, political leaders used pandemic-related fear and anger to promote anti-democratic agendas (Morabia, 2021). Recent research has identified a set of factors that may have promoted such radical developments. These ranged from greater acceptance of conspiracy theories (Levinsson et al., 2021) to an increase in online activities that offered the opportunity to spread misinformation at a fast rate (Davies, 2021). In the current work, we focus on another factor that may have promoted radicalism: feelings of social exclusion. Specifically, we aimed to investigate whether social exclusion that people experienced when they were hit by measures to contain the spread of COVID-19 was associated with higher radicalism.

Social exclusion, being kept apart from others physically or emotionally (Riva and Eck, 2016), is usually accompanied by tremendous psychological stress. Whether exclusion occurs in its indirect form of being ignored by others (i.e., ostracism) or is communicated *via* a direct rejection (see Wesselmann et al., 2016a), the resulting stress manifests in a broad spectrum of consequences. This ranges from a deprived sense of basic needs for belonging, self-esteem, control, and meaningful existence (e.g., Zadro et al., 2004), over a decline in cognitive performances such as effortful logic (e.g., Baumeister et al., 2002), to an activation of neural responses similar to those of physical pain (e.g., Eisenberger et al., 2003). In subsequent behavioral responses, social exclusion makes its victims show both prosocial (e.g., DeWall, 2010) and antisocial behaviors (e.g., Warburton et al., 2006). How subsequent behavior is shaped probably depends on the need that has been threatened predominantly: According to the need fortification rationale (Williams, 2009), prosocial behaviors may fortify inclusionary needs (belonging and self-esteem) as they can achieve re-inclusion; antisocial behaviors may fortify power/provocation needs (control and meaningful existence) as they can provoke power over others and acknowledgment.

Notably, social exclusion has also been associated with radicalism: Previous studies have shown that feelings of exclusion promoted the willingness to identify with and support extreme or radical organizations (Bäck et al., 2018; Hales and Williams, 2018; Renström et al., 2020) and even increased the willingness to commit violent acts on behalf of a radical group (Pfundmair, 2019; Pfundmair and Mahr, 2022). Generally speaking, social exclusion seems to be a condition that can make radicalism flourish (for a review, see Pfundmair et al., 2022). Why excluded individuals increase their openness to radicalism is a question that has only been addressed by few studies. In one study (Knapton et al., 2015), political actions in response to exclusion were mediated by a cluster of needs for belonging and self-esteem. In another study (Pfundmair, 2019), the exclusion-radicalism link was driven by the deprived need for control.

Feelings of social exclusion also rose during the COVID-19 pandemic due to enacted self-isolation (e.g., Killgore et al., 2020; Horigian et al., 2021). Social distancing measures that were used to limit the spread of COVID-19 specifically threatened basic needs for belonging, self-esteem, control, and meaningful existence (Graupmann and Pfundmair, 2022). At the same time, protests against national responses to anti-COVID-19 measures rose worldwide (see, e.g., BBC, 2021). Even acts of violence occurred, for example, when a clerk was shot after asking a customer to wear a face mask (e.g., DW, 2021). Because social exclusion generally has the power to induce radical attitudes and behaviors, it might also have been feelings of social exclusion that contributed to radicalism during COVID-19.

The current study aimed to investigate this assumption. Specifically, we hypothesized that feelings of exclusion induced by measures to contain the spread of COVID-19 were associated with radicalism intentions to illegally and violently fight COVID-19-related regulations (Hypothesis 1). Moreover, we aimed to explore whether this exclusion-radicalism link served to recover basic needs usually threatened by social exclusion. Since previous research did not provide a clear answer to which of those needs might be most relevant in this context, we decided to test each of them. Therefore, we proposed that radicalism intentions against COVID-19-related regulations would fortify one or more of the needs deprived by social exclusion (Hypothesis 2). Notably, we only investigated people who rather opposed the measures to contain the spread of COVID-19 because radicalism intentions to fight those could plausibly only emerge among them.

## Method

### Participants

Following the planned protocol for the more complex analysis (Hypothesis 2), we consulted Fritz and MacKinnon’s (2007) recommendations to determine an adequate sample size for detecting a mediated effect. With an estimated small to medium effect, we followed their suggestions of a total sample size of 148 for a power of 0.80 and alpha and beta levels set at 0.26 using bias-corrected bootstrap tests. We aimed for additional participants to compensate for potential dropouts.

A total of 571 participants started the online study. These were recruited from various German and Austrian research platforms and took part voluntarily. Because we were only interested in people who rather opposed the measures to contain the spread of COVID-19, only those participants who indicated such an opinion were able to continue the study; all other participants were led to the final page of the questionnaire. The former group were 171 participants (30% of the total sample; 101 female, 56 male, 1 diverse, 13 no indication; mean age = 21.82 years, *SD* = 26.74; 137 German, 14 Austrian, 4 other, 16 no indication of nationality).

Collection period was from December 2021 to March 2022. During this time, in both Germany and Austria, most places required proof of full vaccination with a COVID-19 vaccine, proof of recovery from COVID-19, or proof of a negative antigen test (or, depending on the pandemic situation, only the former two were accepted). Notably, Austria additionally established mandatory vaccinations as of February 2022.

### Procedure and materials

After informed consent was obtained, participants were asked to indicate whether they rather opposed or supported the measures to contain the spread of COVID-19. This was used as a filter item; only such participants who indicated a rather opposing opinion could continue the questionnaire. Then, they were asked how socially excluded and how deprived in their basic needs they felt due to the measures to contain the spread of COVID-19. After that, we assessed their radicalism intentions as well as explicit items to depict their intentions to fight for their opinions. Then, participants indicated their political opinion and sociodemographic data. At the end, they were thoroughly debriefed.

#### Opinion about COVID-19 measures

Participants were asked to indicate whether they were in favor or against the current measures to contain the spread of COVID-19. They indicated their level of agreement on a 1 = *strongly oppose it*, 9 = *strongly support it* response scale. Only those participants who indicated ≤4 could continue the study.

#### Feelings of social exclusion

To assess how much the state measures that were taken to contain the spread of COVID-19 affected the participants’ feelings of social exclusion, we provided three items. In the first item, we focused on social exclusion in a more general term; in the second and third item, we focused on specific subtypes of exclusion, viz. ostracism and rejection. Thus, on a scale from 1 = *not at all* to 7 = *very much*, participants responded to the following items: “How strongly do you feel excluded/ignored/rejected due to the COVID-19 measures?” Although theoretically different, all three items intercorrelated highly, *r*s between 0.72 and 0.82. Therefore, we combined them to an overall exclusion index (α = 0.91).

#### Deprivation of basic needs

To assess the basic needs regularly deprived by social exclusion, participants responded to a 4-item needs-threat short scale (Rudert and Greifeneder, 2016). They were provided four items to answer the question of how they felt due to the COVID-19 measures. Using 7-point semantic differentials, they assessed their levels of belonging (“rejected-accepted”), self-esteem (“devalued-valued”), control (“powerless-powerful”), and meaningful existence (“invisible-recognized”).

#### Radicalism intentions

Participants’ radicalism intentions were assessed using the Activism and Radicalism Intention Scale (ARIS; Moskalenko and McCauley, 2009). Participants were instructed to indicate how much they would engage for their opinion on how to deal with the pandemic. They responded to four items of the activism intention subscale pertaining to non-violent and legal behaviors (e.g., “I would donate money to an organization that fights for my opinion about how to deal with the pandemic,” α = 0.88) and four items of the radicalism intention subscale pertaining to illegal and violent behaviors (e.g., “I would continue to support an organization that fights for my opinion about how to deal with the pandemic even if the organization sometimes breaks the law,” α = 0.89) (Notably, data on the former subscale was collected for the sake of completeness but was not included in the main analyses.). Each item was completed on a 1 = *disagree completely* to 7 = *agree completely* scale.

To check validity of the ARIS scale, we also assessed six individual items to test which measures participants would take to fight for their opinion about how to deal with the pandemic. On 1 = *not at all* to 7 = *very much* response scales, they assessed whether they would not at all fight for it, join a group with the same opinion, perform non-violent acts, accept property damage, threaten people with violence, or accept personal damage.

#### Political opinion

Participants were asked to map their political opinion on a scale ranging from 1 = *left* to 10 = *right* (Breyer, 2015).

## Results

### Preliminary results

To check validity of our radicalism scale, we explored how much activism and radicalism intentions correlated with the explicit measures participants would take to fight for their opinion. These correlations are presented in Table 1. Supporting the scale’s validity, activism correlated most strongly with the willingness to join a group with the same opinion and to perform non-violent acts, whereas radicalism correlated most strongly with the willingness to threaten people with violence and accept property damage.

**TABLE 1:** Correlations among activism and radicalism items.

	No action	Joining a group	Non-violent action	Accepting property damage	Threat of violence	Accepting personal damage
Activism	–0.18*	0.71***	0.56***	0.51***	0.40***	0.36***
Radicalism	–0.15	0.31***	0.15	0.67***	0.74***	0.66***

*p < 0.05, ***p < 0.001.

Moreover, to gain an overall impression of the sample’s general intentions for radicalism, we conducted frequency analyses. Participants who indicated the highest levels (= scale point of 7) of radicalism were only a fraction of the whole sample: 2% (*n* = 3). This matched the sample’s low mean value of the radicalism scale (*M* = 1.90). Accordingly, the skewness of radicalism was found to be 1.88, indicating that the distribution was right-skewed, while still normal (e.g., Hair et al., 2010).

Intercorrelations between all analyzed variables can be found in Table 2. Consistent with other research (see Wesselmann et al., 2016b), these revealed large intercorrelations among the basic needs as well as medium to large correlations between the needs and the social exclusion index. Although clearly related, the correlation indices did not indicate a complete overlap, supporting the assumption that they are conceptually distinct. It is also worth noting that the intercorrelations revealed a (plausible) negative relationship between the participants’ agreement with containment measures and social exclusion as well as radicalism and a positive relationship between the participants’ agreement with containment measures and fulfilment of most of the basic needs.

**TABLE 2:** Means (and standard deviations) of as well as intercorrelations between analyzed variables.

	*M (SD)*	(1)	(2)	(3)	(4)	(5)	(6)	(7)
(1) Social exclusion index	4.85 (1.72)	—						
(2) Belonging	2.71 (1.43)	−0.62***	—					
(3) Self-esteem	2.50 (1.40)	−0.57***	0.79***	—				
(4) Control	1.93 (1.34)	−0.42***	0.61***	0.65***	—			
(5) Meaningful existence	2.47 (1.47)	−0.41***	0.62***	0.63***	0.66***	—		
(6) Radicalism	1.90 (1.33)	0.17*	–0.09	–0.06	0.13	0.03	—	
(7) Political opinion	4.72 (1.79)	0.09	–0.01	–0.05	0.07	0.02	–0.12	—
(8) Agreement with containment measures	2.77 (1.02)	−0.37***	0.28***	0.34***	0.17*	0.09	−0.25**	–0.01

***p < 0.001; **p < 0.001; *p < 0.05.

### Testing hypothesis 1: The link between exclusion and radicalism

To investigate whether feelings of exclusion induced by measures to contain the spread of COVID-19 were associated with radicalism intentions to illegally and violently fight COVID-19-related regulations, we conducted a regression analysis. Because radicalism is known to vary by age, gender, and political ideology (see, for example, Chermak and Gruenewald, 2015), we tested the link between exclusion and radicalism while including those as control variables. We also added nationality as control variable because COVID-19 restrictions in Germany and Austria during that time were similar but not identical. Therefore, we conducted a two-stage hierarchical multiple regression with radicalism as the dependent variable; age, gender, political orientation and nationality were entered at stage one and the exclusion index was entered at stage two.

**FIGURE 1:**
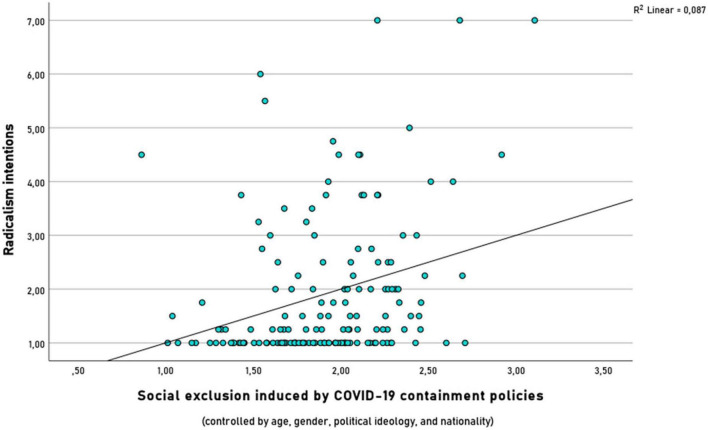
Results of the regression model: Feelings of social exclusion induced by the COVID-19 containment policies predicted radicalism intentions to illegally and violently fight against those; the regression model controlled for age, gender, political ideology, and nationality.

The regression analysis revealed that at stage one, age, gender, political orientation and nationality did not significantly contribute to the regression model, *F*(4,154) = 1.43, *p* = 0.228, accounting for 3.7% of the variation in radicalism. Adding social exclusion to the regression model explained an additional 5.1% of variation in radicalism and this change in *R*^2^ was significant, *F*(5,154) = 2.85, *p* = 0.017. Thus, feelings of social exclusion induced by COVID-19 containment policies revealed to be a meaningful predictor for radicalism intentions, see Figure 1.

### Testing hypothesis 2: Favorable consequences of the exclusion-radicalism link

To investigate whether radicalism fortified needs deprived by social exclusion, we conducted mediation analyses using the Process tool by Hayes (2013; model 4, 5,000 bootstrap samples). We entered the exclusion index as independent variable, radicalism as mediator, and feelings of belonging, self-esteem, control, and meaningful existence as separate dependent variables. As in the former analysis, we also included age, gender, political ideology, and nationality as covariates.

**FIGURE 2:**
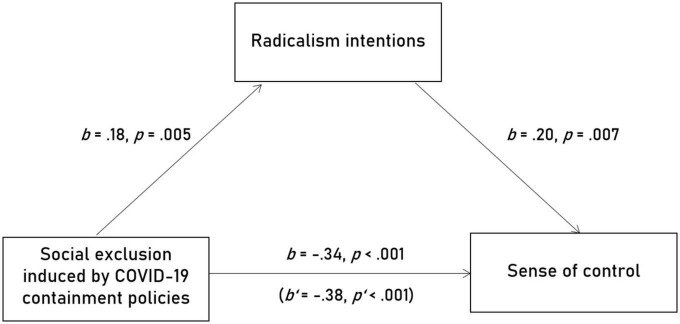
Results of the mediation analyses: Feelings of exclusion that were *per se* associated with lower control increased radicalism. In turn, radicalism translated to a perceived increase of control.

The total effect revealed to be significant for all needs, belonging: *b* = –0.53, *SE* = 0.05, *t*(149) = –9.79, *p* < 0.001, self-esteem: *b* = –0.45, *SE* = 0.06, *t*(149) = –7.76, *p* < 0.001, control: *b* = –0.34, *SE* = 0.06, *t*(149) = –6.53, *p* < 0.001, meaningful existence: *b* = –0.38, *SE* = 0.06, *t*(149) = –6.12, *p* < 0.001. This replicates the well-known negative relationship between feelings of social exclusion and fulfilment of individual needs. In a next step, this relationship was decomposed into a direct link and an indirect link (i.e., transmitted through the mediator). The direct effect was also significant, belonging: *b* = –0.54, *SE* = 0.06, *t*(149) = –9.55, *p* < 0.001, self-esteem: *b* = –0.45, *SE* = 0.06, *t*(149) = –7.76, *p* < 0.001, control: *b* = –0.38, *SE* = 0.06, *t*(149) = –6.53, *p* < 0.001, meaningful existence: *b* = –0.40, *SE* = 0.06, *t*(149) = –6.34, *p* < 0.001. For belonging, self-esteem, and meaningful existence, the indirect effect was not statistically different from zero as evidenced by bootstrap confidence intervals that contained zero, belonging: *b* = 0.003, *SE* = 0.01, 95% CI = [–0.02, 0.02], self-esteem: *b* = 0.003, *SE* = 0.01, 95% CI = [–0.02, 0.02], meaningful existence: *b* = 0.02, *SE* = 0.01, 95% CI = [–0.001, 0.05]. However, for control, it was, *b* = 0.04, *SE* = 0.02, 95% CI = [0.01, 0.07]. That is, feelings of exclusion that were *per se* associated with lower control increased radicalism and radicalism, in turn, translated to a perceived increase of control. The path coefficients of this mediated effect are plotted in Figure 2. This regression model explained 27.8% of variation.

### Additional analyses: Moderation by political opinion

To check whether the participants’ political opinion influenced the current effects, we conducted additional analyses.

First of all, the political opinion was relatively equally distributed in our sample: rather left (= scale point between 1 and 4): *n* = 64, middle (= scale point of 5): *n* = 42, rather right (= scale point between 6 and 10): *n* = 53.

To investigate whether political opinion moderated the exclusion-radicalism link, we conducted a moderator analysis using the Process tool by Hayes (2013; model 1, 5,000 bootstrap samples). We entered the exclusion index as independent variable, political opinion as moderator, and radicalism as dependent variable; age, gender, and nationality were included as covariates. The regression model revealed a significant main effect of exclusion, *b* = 0.40, *SE* = 0.16, *t*(148) = 2.55, *p* = 0.012, replicating the known positive relationship between feelings of social exclusion and radicalism intentions. However, no significant main effect of political opinion, *b* = 0.14, *SE* = 0.17, *t*(148) = 0.83, *p* = 0.409, and no significant interaction effect emerged,*b* = –0.05, *SE* = 0.03, *t*(148) = –1.52, *p* = 0.130. Thus, the exclusion-radicalism link was not driven by one political subsample but, instead, existed in both more leftist and more rightist participants.

## Discussion

The current study aimed to investigate feelings of exclusion induced by measures to contain the spread of COVID-19 and whether these were associated with radicalism intentions to illegally and violently fight COVID-19-related regulations. Studying a sample of individuals who opposed the measures to contain the spread of COVID-19, we indeed found evidence for this link, which supported Hypothesis 1. This not only replicated the exclusion-radicalism link observed in experimental settings (e.g., Hales and Williams, 2018; Pfundmair, 2019; Renström et al., 2020) but added one more factor that may have promoted radical developments in the COVID-19 crisis.

A secondary aim of the current work was to investigate whether radicalism intentions against COVID-19-related regulations served to fortify needs that are usually threatened by social exclusion. We also found evidence for this claim, supporting Hypothesis 2. We specifically identified the need for control as a relevant factor in the exclusion-radicalism link, similar to previous experimental work (Pfundmair, 2019). In other words, the relationship between exclusion and radicalism was associated with recovering a sense of power. This pattern appears plausible when consulting the radicalization literature: Re-establishing a sense of certainty, a type of predictive control, is known to be one catalyzer for radicalization (see Hogg, 2014).

In additional exploratory analyses, we found the political opinion to be unrelated to the found pattern. Thus, the association between exclusion and radicalism emerged in both more leftist and more rightist individuals. This fits the observation that protests against the COVID-19 measures were approved by people of diverse political backgrounds, although the far-right was most dominant (e.g., Plümper et al., 2021). Moreover, in experimental work investigating the exclusion-radicalism link, ideology did not appear to play a moderating role (Renström et al., 2020).

Notably, in the current study, we investigated how much the state measures that were taken to contain the spread of COVID-19 affected the participants’ feelings of social exclusion. That is, we measured a very specific form of exclusion which fleshed out as both limited personal interactions and political restrictions enforced against the participants’ personal convictions. Previous research has already shown that social exclusion relating to larger-scale incidents in societal contexts (e.g., structural-societal conditions, politics) is also able to induce individual feelings of social exclusion. For example, women’s psychological reactions to female underrepresentation in male-dominated academic fields mirrored those typically induced by interpersonal instances of exclusion (i.e., a threat to fundamental needs; McCarty et al., 2020). Another study showed that having voted for a losing-side candidate in presidential elections was associated with emotional pain of first-hand experienced social exclusion (Young et al., 2009; Claypool et al., 2020; Salvatore et al., 2021). In cases like these, perpetrators depicting the source of social exclusion are often abstract (e.g., society or the State). This might facilitate aggressive responding because exclusion is suggested to induce aggression whenever there is no adequate source of (re-)gaining acceptance (see DeWall and Bushman, 2011). Radicalism intentions after social exclusion induced by COVID-19 containment policies might fall into the same category. Furthermore, the readiness to engage in illegal and violent political action might be a particularly useful tool in this context because it is in some way directed against the abstract perpetrator, the State.

The current work benefited from its high ecological validity: We investigated people opposing the measures to contain the spread of COVID-19 in the middle of the pandemic. This field approach, however, comes with the limitation that our sample was rather small and our conclusions are correlational. Thus, we do not know whether feelings of exclusion might have induced radicalism or radicalism might have induced exclusion. Indeed, research has not only demonstrated that social exclusion can promote radicalism (e.g., Pfundmair, 2019), but also that individuals holding extreme attitudes are at a higher risk of being excluded by others (Hales and Williams, 2019). Yet, in the current study, there could be a case for the first assumption because we asked for exclusionary feelings due to the COVID-19 measures and not due to other individuals. Moreover, theoretically, our mediation model, which indicated a relationship between exclusion and rebuilding a sense of power *via* radicalism, could also take a different form. Indeed, similar statistical effects emerged when we switched radicalism as a mediator with control. However, in doing so, the model no longer made sense in terms of content: While exclusion was linked to both deprived control and radicalism (the well-known effects), more control led to more radicalism (a relationship not quite comprehensible). Thus, there are reasons to embrace the proposed causality. Nevertheless, the present cross-sectional data cannot ultimately substantiate causality.

The specificity of the current sample should also be noted here. The mean value of the radicalism subscale was relatively low, which indicates that we did not investigate a sample of radicals but tendencies in radical developments. This low level of radicalism in the general population fits observations that radicalized individuals are a rather exceptional phenomenon in a society. Moreover, we only investigated radicalism intentions and not behaviors. Our scale to measure such intentions appeared highly valid since we found high correlations with the willingness to threaten people with violence, to accept property damage, and even with the willingness to accept personal damage. However, it must be considered that radical beliefs, as assessed in this work, must not inevitably lead to radical action, although they can inspire radical action (McCauley and Moskalenko, 2017). Lastly, it should be recalled that we only investigated opponents of COVID-19 measures. It is quite conceivable that increased exclusion also came with increased radicalism among supporters of the measures – then, of course, regarding the topic of how to fight for the enforcement of COVID-19-related regulations. Further, a relationship between exclusion and radicalism naturally also exists outside times of COVID-19, as evidenced by previous research (e.g., Hales and Williams, 2018; Pfundmair, 2019; Renström et al., 2020).

Altogether, the current findings bring along several theoretical implications: First, demonstrating a relationship between feelings of social exclusion and radicalism in one of the newest radical developments (radical attitudes and behaviors emerging in the string of events of the global pandemic), they replicated the exclusion-radicalism link in a field approach. Second, the current findings underline the importance of control as a motivator for radicalism. Strengthening a sense of power through radicalism seemed to help those who opposed the COVID-19 measures to cope with the social pain. A fruitful avenue for future research would be to further investigate the power of powerlessness in this context. In practical terms, on the other hand, knowledge on social exclusion as a risk factor for radicalism (not only but also) during the COVID-19 pandemic might help shape intervention efforts – which might be useful to avoid paying a high political price.

## Data availability statement

Materials and data of this study can be accessed openly at https://osf.io/4HGQR.

## Ethics statement

Ethical review and approval was not required for the study on human participants in accordance with the local legislation and institutional requirements. The patients/participants provided their written informed consent to participate in this study.

## Author contributions

MP performed statistical analyses, interpretation of results, and wrote the original draft. LM provided critical revisions. Both authors developed the study design, collected data, and approved the final version of the manuscript for submission.
